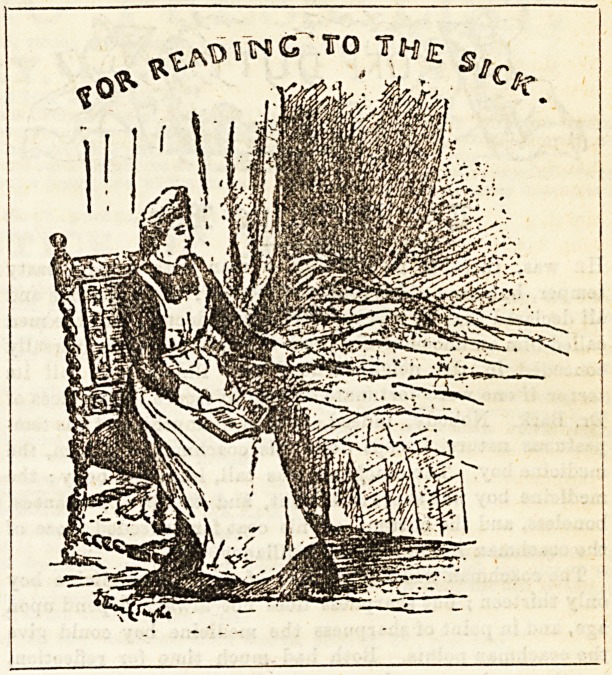# The Hospital Nursing Supplement

**Published:** 1892-07-16

**Authors:** 


"The Hospital" Nursing Mirror.

Being the Extra Nursing Supplement of "The Hospital" Newspaper.

Contributions for this Supplement should be addressed to the Editor, The Hospital, 140, Strand, London, W.C., and should have the word "Nursing" plainly written in left-hand top corner of the envelope.

### En Passant.

MEMORIAL TO NURSE COATES.—The cross which has been put up in Ayr Cemetery to the memory of Nurse Coates, who died last December in the County Infirmary of typhus, is of blue Ayrshire stone, and bears the inscription, "In loving memory of Letitia Coates," with the date of her death and birth. She was only 22 years old, and contracted the fever while in attendance on the typhus patients. The cost of the grave and cross has been given by the directors and friends of the hospital.

ON COMMISSION.—We hear of impecunious folks who make for themselves an uncertain and, generally, a poor living, by selling all kinds of things "on commission." Then there is the more successful and higher stamp of business man, whose income is derived from various sources, but on a similar principle. But we hear now of a, to us, quite new form of commission, viz., one demanded and received by certain medical men who have introduced a nurse to a new case and expect a percentage on her fees. Surely this novel departure is limited to but a small and inferior section of the most liberal of all professions.

SYDNEY HOSPITAL.—The nurses at this hospital have taken up their residence at the old Australian Club House as the Nightingale Home, their own particular abode is now in the temporary possession of the inmates of the old wooden pavilion now doomed to destruction as soon as the permanent enlargements to the hospital are completed. Miss Gould, the present Matron, has succeeded Miss McKay nearly a year now and during the year twenty-four probationers have been taken into training, of whom eighteen remain. Two nurses were appointed matrons of county hospitals, two were appointed head nurses at county hospitals, and two have taken up private nursing. These few appointments from one single Australian hospital will show many of our correspondents that we are right when we say that it is foolishness for any nurse to go out to the colonies on mere chance unless she can afford the risk of probably a long wait. Things develop quickly now-a-days, and the colonies are fast finding that local talents will keep the supply equal to the demand for trained nurses.

CHRISTMAS COMPETITIONS.—We hope this year to have as many well-made garments for distribution among the adults in the hospitals at Christmas as we had this past foggy season, and so we mention our Christmas parcels now, in the hope that some of our kind readers may fill in a few spare moments of their holidays by stitching for us. This Christmas we will give the same prizes as last year in either books or money as the winners choose: (1) For the best pair of socks knitted by a nurse, 5s.; (2) for the best pair of socks knitted by any Hospital reader, 5s.; (3) for the best made flannel shirt, 10s.; (4) for the best made woman's blouse, 10s.; (5) for the best made flannel petticoat, 10s.; (6) for the best made and best shaped dressing gown for an invalid cut out and made by a nurse, 20s. It will be seen that Nos. 1 and 6 are reserved for nurses only. Flannelette is cheap, and light, and warm, and would therefore form the best material for the dressing-gown. In judging, four marks are given for workmanship, four for shape, and two for general appearance; therefore, it is not wise to spend time on elaborate trimmings. Long seams may be done by machine.

ANOTHER'S REPUTATION.—In a woman who has mastered the art of nursing, as well as the science of medicine, we ought to find a very perfect personage indeed. The most sympathetic and pleasing of the lady-doctors are those who acknowledge and understand the value of skilled nursing. Speaking at the School of Medicine for Women the other day, Mrs. Peachey Phipson gave the students some advice which our nurses might well take a lesson from. Referring to the days when mud was literally thrown at the pioneers of the present race of students, she warned these latter, whilst congratulating them on their smoother path to knowledge, that no mud fails in staining—some of it sticks. Thinking of these words, spoken calmly by a successful woman, all may well reflect and check the words which may stain another's reputation, either directly or indirectly.

THE SOCIETY FOR THE EMPLOYMENT OF WOMEN.—This society is now in its thirty-fourth year of work, and goes on its way doing good every day. During the past year 73 persons have been found permanent employment, and on 593 occasions temporary work has been found, while 79 girls are undergoing special technical teaching through the agency of the society. The register for those seeking employment receives names free of charge. Now that the question of restriction of women's labour is so much before the public, the society has appointed a sub-committee to keep a watchful eye over the right of women to labour, and to prevent any restrictions being made which are not also applied to men. It is also owing to the efforts of this society that the idea of women as factory inspectors was first started. We should like to see the funds of this modest society augmented; for those who can afford to give, it is a good opportunity for women to stretch out their hand to women.

KENSINGTON DISTRICT NURSING ASSOCIATION.—The annual meeting of this Association was held on Friday, July 1st, by the kind permission of Mr. and Mrs. W. C. Alexander, at Aubrey House, Campden Hill, when the President of the Association, H.R.H. Princess Louise, Marchioness of Lorne, was present. Among those present were the Rev. the Hon. E. C. Glyn and Lady Mary Glyn, Lady Millais, Miss Louisa Twining, Mr. Bousfield, Mr. Ellis, Mr. T. C. Horsley, R.A., Mr. F. D. Mocatta, and other friends of the Association. Mr. Carr Glyn took the chair. Mr. Thicknesse (hon. treasurer) read the annual report, which was to the effect that as the work was increasing new subscriptions were urgently needed. The number of visits paid by the seven nurses last year was nearly 20,000, as against 18,000 the year before. The Chairman then made a short appeal for more subscriptions, and said that it would be a severe blow to Kensington if the nursing staff had to be reduced. He then called upon Mr. Barker, of Harley Street, who, in moving the adoption of the report, paid a hearty tribute to the usefulness of district nursing. Miss Louisa Twining seconded the resolution, saying that it would be a disgrace to Kensington if the work was allowed to languish for lack of funds. These district nurses, who nursed the poor in their own homes, were now to be found all over England, and she described what had been done at Worthing under her influence. Mrs. Franks (who used to be the head of the Kensington Infirmary, and was introduced as the hon. secretary of the Haggerston and Hoxton District Nursing Association) described in detail a district nurse's day, giving some amusing incidents experienced in the work, and pointing out how it educated the poorer classes in habits of cleanliness and better sanitation. During the afternoon each nurse on the staff was presented by the Princess with the bronze medal of the Queen Victoria Jubilee Institute of Nurses.

### Ventilation, Disinfection, and Diet.

By P. Caldwell Smith, M.D.

### XIV.—DIET AND DIETARIES.

Pork and Bacon—Poultry—Game — Rabbit—Hare—Fish—Cooking of Different Kinds of Meat—Beef Tea—Danger of Eating Raw Meat—Economy in Meat Diet—Eggs.

It is not to be wondered that pork and bacon have always been a favourite dish, especially among the working and specially the agricultural classes. The pig some years ago was a necessary concomitant of the agricultural labourer's three acres and a cow; and this arose from the fact that its meat is nutritious, and that the animal is easily reared. The ease with which pig's flesh may be preserved has also something to do with its common use. There is a larger amount of fat in the pig as compare the percentage composition. In the pig nitrogenous matter is 14·5 per cent., fat 37·34; while in beef (ordinary), N. = 21 per cent., fat = 6 per cent.; while in the fattest beef the average amount is 27 per cent. Pork is, of course, much more difficult of digestion, roast pork taking about five hours. When cured or pickled it is more digestible, young cured pork being probably digested in from three to three and a-half hours. It is not a food to be recommended to invalids, but is chiefly one for those in robust health with good digestions. If taken at all it should be thoroughly masticated before being swallowed, so that the fibres of the meat part may be well broken up. It is also not a food to be given to children under six or seven years, as not only do they chew very quickly, but the digestive juices are not powerful enough to prepare the food for being taken into the system.

Bacon is the term applied to the sides of the pig which have been removed and preserved with salt and saltpetre. After being well rubbed with this for about three weeks it is dried by means of wood smoke. It is rather more digestible than pork or the leg part of the pig, while, owing to its agreeable flavour, it is much used both by rich and poor.

A large amount of poultry and game is consumed in this country, but these, although very useful as invalid's food, are not sufficient for continuous use in the case of a healthy working adult. This arises from the fact that fowls contain a very small proportion of fat, only about 3 per cent. This is recognised by all cooks, so it is quite customary to serve bacon of some kind with fowl to make up for this deficiency. Again, the flavour of fowl soon palls on one, and no one could possibly take it every day. The different kind of birds differ very largely with respect to the quality of the flesh. This is in game much firmer and harder, and one reason why pheasants, grouse, &c., are not eaten till they become what is called high is that the slight decomposition permitted causes separation of the tough fibres and consequently makes them more digestible.

The flesh of the rabbit very soon induces disgust if taken too frequently. It is certainly easily digested, and should be used, perhaps, oftener than it is during convalescence. The flesh of the hare is more difficult of digestion, and although hare soup may be taken by invalids, yet the flesh is too strong and indigestible.

Fish is one of the most useful articles of diet which we have. It is used now very largely used by the middle classes as an ordinary article of diet, but among the working classes it is not used to the extent it should. The only exception to this is in regard to the herring, which is used both fresh and salted in large quantities by the working class, but they avoid fresh fish far too much. The number of fish which are used is very large, the more common being codling, sole, haddock, whiting, herring, halibut, salmon trout. The amount of nitrogenous matter in these varies very much, fresh herrings containing only 10 per cent., while the skate contains 24 per cent. The amount of fat also varies from 25 in the sole to 16 in salt herrings. Whiting, which is a very favourite and delicate fish for the use of invalids and convalescents, contains about 15 per cent. of nitrogenous and only 38 per cent. of fat, sole containing even a smaller amount of both these constituents. These two fish should enter largely into the diet of invalids.

In regard to the cooking of the different kinds of meat it is always to be remembered that if it is to be eaten, the juices should be, as far as possible, kept in the meat. In roasting meat this is done by the heat coagulating the albumen on the surface, and thus keeping the juice from escaping. In boiling meat, if the meat only is to be used, and not the water in which it is boiled, it should first be immersed in boiling water, so that the albumen is coagulated as in roasting. The meat should then be allowed to remain in the boiling water for five or ten minutes cold water can then be added, and the cooking gone on with. If beef tea is to be made, the very opposite of this has to be done, as you wish to extract as much as possible from the meat. Liebig says, "Finely minced meat should be extracted with its own weight of cold or luke-warm water, digested for an hour, all the albumen and the other soluble ingredients will be removed into the tea. On straining through a sieve and slight washing with water, the beef tea may be heated, flavoured, and served." Fish is cooked in a variety of ways, boiling and frying being the most common. All fish should be taken as fresh as possible, although some authorities think that the flavour is increased by being kept for 24 hours.

A word regarding economy in meat diet. A piece of steak is really a less expensive portion of meat than a joint, as it contains no bone, even although the former is sold at 10d. or 1s., and the latter at 1s. 2d. or 1s. 3d. Comparing this with cod, we find that it is much more economical than meat. One pennyworth of good beef contains only 1¼ oz., of which ⅞ of an ounce is water; while one pennyworth of cod, when it is 3d. the lb., would contain 5⅓ ounces, of which 4 ounces is water. Dr. Wynter Blyth, in speaking of this, says: "What is true of cod is also true of all the cheaper white fish; directly the price of any white fish sinks to 2d. or 3d. the pound, the advantage over the joints of the butcher is evident. There is no more practical way of cheapening food for the hungry classes than by encouraging the fishing industries, facilitating the transit of fish from the coast inland, establishing markets, and lastly, teaching the people how to cook their fish properly."

One of the most important foods we have is the egg. The egg of any bird can be eaten by human beings, as they have all pretty much the same composition, although they differ largely in flavour, from the egg of the ordinary hen to one of the strongest known—viz., that of the seagull. The egg, as you know, is composed of a yolk in the centre surrounded by the white. The white of the egg consists of almost pure albumen with water, while the yolk contains also sulphur, and an oil rich in phosphoric acid. An ordinary hen's egg weighs from one and a-half to two ounces, while that of a duck weighs from two to three ounces, and of a goose four to six ounces. Eggs are taken, as you know, in many different ways—soft boiled, hard boiled, raw, poached, in form of omelets or soups, and with milk as custards, &c. Eggs are more digestible when raw, or soft boiled, as the albumen is not so dense. Eggs are also given with wine or brandy, the former being, perhaps, the more easily digested.

The poached egg, as made in France and the East, is a very agreeable form of serving, especially to invalids. A pottery dish is used with a charcoal fire, and the heat consequently kept moderate. The dish is very thick, so that it takes some time to heat. Butter, pepper, and salt are then placed in it, the egg broken, and dropped into it. The yolk should not be broken, and it should be turned so that both sides are slightly browned. Fresh eggs are more digestible than those which have been kept. Eggs take from three to four hours to digest, as has been proved by Dr. Beaumont's experiments.

### A Plan of Instruction in Invalid Cooking for Training Schools.

By ISABEL A. HAMPTON, Superintendent of Nurses, and Principal of the Training School of the Johns Hopkins Hospital.

THE knowledge of how to prepare food suitable for sick people, and the kind of food necessary for invalids, is a branch of nursing that the majority of schools for nurses leave chiefly to their pupil's intuition to evolve, and a more necessary or important branch to teach can hardly be named. For is it not true that a nurse must recognize the importance of carefully and properly nourishing her patient, as well as knowing how to give baths, reduce temperatures, care for beds, administer medicines, and the many things that a nurse must know about illness of any kind? And yet, we who are practically concerned in training nurses, would decline to organise a school upon theoretical instruction solely. While we consider the theory of nursing necessary in a course of a nurse's instruction, we all maintain that it is next to valueless without the addition of the daily practical work; and that this practical work is the important feature in training nurses. It is one thing to tell a woman how a bed should be made for an invalid and another to show her how, and have her do it under observation and criticism day after day until she can do it perfectly. And if this is so important in one branch of nursing, should it not hold equally good and be as equally important to carry it into each department of her work if you would have a well equipped, intelligent nurse when her probation work is over? On the contrary, so far as the special teaching regarding the diet for sick people has been given it has been chiefly theoretical, and has been told by the lecturer on dietetics. Anything in the way of practical teaching has been carried on through the medium of outside cooking schools, where pupils in small classes could be sent once or twice a week for two hours for a course of from twelve to eighteen lessons. Another plan is to have some teacher from a cooking school come to the hospital once or twice a week for twelve or fifteen lessons, and give lectures and demonstrations to classes of nurses. This is rather better than nothing, but the results are of little practical value, the difficulty being to put such lectures into practice under the direction and observation of a teacher, especially as it is the exception rather than the rule for women of the present day to be *au fait* with the management of a household, including the culinary department. With the women who come to training schools, we are very apt to be obliged to begin at first principles with them, in regard to order, cleanliness, system, &c., and so it makes it so much more important that our methods of instruction should be as thoroughly practical as possible in all particulars, and especially in this matter of diet, if we would avoid having our patient's convalescence retarded by inappropriate or ill-prepared food.

In attempting to demonstrate a plan for the practical instruction of invalid cookery, it is not our idea to make cooks of nurses, for more often than not a nurse has not the time to prepare her patient's food, but it is that she may have an intelligent idea of what manner of food the patient ought to have; the best and most hygienic methods of preparing it, and to tell how it should be prepared; to have a varied and extensive list at her command for variety's sake, and the more æsthetical mode of serving. If she is a district nurse to be able to tell the mothers of families she visits how they can spend a small sum for food in the most profitable manner and give valuable hints and suggestions as to the manner of preparing it. With such objects as these in view the authorities of the Johns Hopkins Hospital granted to their department of nurses permission to open a cooking school in the hospital for the instruction of their pupil nurses.

DIET KITCHEN OF THE JOHNS HOPKINS HOSPITAL TRAINING SCHOOL FOR NURSES, BALTIMORE.

The endeavour has been from its beginning to have it on as economical a basis as possible.

The chief requisites as well as the chief expenses have been a competent teacher, and a well equipped kitchen; but the expense of the latter can be partially met by small gifts of necessary china, trays, cooking utensils, &c., from ladies interested in the school. A room for kitchen purposes should be situated at an equally convenient distance from all the wards as possible; it is not necessary to have a large room, one 12 ft. by 20 ft. is sufficient. It should be fitted up with hot and cold water, sinks, dressers, and a small icebox. A good teacher is indispensable. She should be well up in practical chemistry, clear in her statements, clean, and methodical. The course of instruction has been increased from one month to six weeks, the last fortnight being especially devoted to instruction for district nursing purposes. As the primary idea in establishing the school is "to teach," the obligation to prepare dishes for hospital use is secondary, and only a few and special orders are filled daily for patients who cannot eat what is ordered from the general hospital kitchen, but all the dishes prepared are sent into the wards to be distributed at the discretion of the ward head nurse. The only exception to this rule is the beef tea, chicken broth, and a portion of the oatmeal porridge, which are prepared daily for the entire hospital.

*(To be continued.)*

### Sisters.

We do not propose to deal with any but Ward-Sisters in this article, for though religious sisterhoods still undertake the nursing of one or two hospitals, and also do good work amongst the poor lying sick in their own homes, yet they hardly come within our present intention of treating the various grades in what may be described as unsectarian nursing. The Ward Sister holds a somewhat unique position, which is, however, a very pleasant one, as the affection and confidence she can win and the influence she may exercise are practically unlimited, and so long as she keeps the rules of her institution, and respects the wishes of the committee and the staff, she is pretty sure to receive the approbation and support of the Matron. The work and remuneration of Sisters vary greatly in different hospitals, and perhaps the last is not always in due proportion to the first. As regards allotment of the nurses, much is left to the Sister's judgment; that is to say, a daily routine has to be observed, and certain duties have to be got through by a limited number of people, and, except for traditions of usage, she is pretty free to make what seems to her the best arrangement in her power, just as the mistress of a private establishment might do, and in cases where she is a woman of mature experience, being also a thoroughly trained nurse, she is perfectly capable of fitting in these details justly.

In some hospitals there now exists an excellent plan of advancing nurses to be "probationer-sisters," to work with, and under, the regular established heads of wards, and thus they become thoroughly conversant with all the duties attached to the post before they are called upon to take up for themselves the responsibility also. The position of Ward Sister is often a more enviable one than that of a Matron, as many a woman has discovered when she has left the former for the latter appointment. As a lady who is proving herself a most competent Matron said the other day, "When I was a Sister all my nurses believed in my good intentions, and accredited me with a knowledge of my duties, as a matter of course; but now every one, both within and without the hospital, feels perfectly free to criticise 'the Matron' without mercy." It is sometimes the custom to make the lady pupils or paying probationers into Sisters at the end of one year's training, which is assuredly an unwise proceeding, for no amount of previous education can fit a person who has had such short ward experience to rank over staff nurses, nor yet to instruct probationers properly. The Sisters in themselves afford a study full of infinite variety, for there are still a few examples of the old school amongst us, and some of these are excellent nurses, but by no means all of them, for when sufficient intelligence to keep pace with the times is absent, these women are apt to remain fixed in the ways and practices which obtained favour in the days of "untrained" labourers. However, if they are good and faithful members of the hospital staff, they will probably remain attached to it until they reach a hale old age, and are entitled to a pension. Some of them are veritable evergreens, who, during a quarter of a century perhaps, have watched the rise and fall of many brighter stars, young and cultured women who have gone to illumine fresh fields, being in their turn succeeded by others, whose half-a-dozen years of energetic work are looked upon with a sort of tolerant indulgence by these older members of the Sisterhood! From this younger race much more is required, both of knowledge and ability, than served for the generation which is fast becoming obsolete, and we cannot specially commend the ornamental type whose appearance and manners may be excellent, but her experience is elementary, and she has no capacity for conveying instruction, whilst in an emergency she promptly summonses help, instead of giving the personal assistance which the true nursing sister instinctively bestows. Still, this lady may have a clear conception of discipline, and possibly, after all, she manages a small ward "indifferent well"! The muddling Sister who neither works well herself, nor yet abstains from hindering her better-qualified subordinates, is a most trying personage, although she may be possessed of a kind heart which leads her friends to say apologetically "Poor thing, she means well;" and doubtless she does, and she never earns that active dislike which is accorded to the strict disciplinarian. The latter is present in every hospital, and has usually gone through a long and complete "training," which has, unfortunately, grafted on to her naturally hard disposition additional sternness; she never excuses any shortcomings on either her own or her nurses' parts; she is dreaded by probationers and by the majority of the nurses who come in contact with her, although an occasional exception is made by some steady, hard worker, who recognises and respects in her superior the same conscientiousness which directs her own conduct. There is a large array of incompetent Sisters, some few of whom may be individually good nurses, but quite unfitted for supervising others; but, on the other hand, there are, happily, scores of capable women in charge of hospital and infirmary wards, and these reign supreme in the hearts of their patients, as well as earning respect and obedience from their nurses, whilst the probationers indulge in even more unmitigated admiration. These wise and kindly women, whose training and experience ensure consideration from the cleverest of doctors as well as from the rawest of students, owe much of their influence to their invariable courtesy to all; being faithful and honest themselves, they count on the presence of equal integrity in others, and they frequently obtain that which they trust in. Judged by probationers, the lot of a Sister appears an easy one; they see the respect paid to her, and the superiority of her position, but they generally fail to realise the many occasions on which she is forced to serve as a "buffer" between their blunders and possible consequences to themselves and others, and they forget that she is really only one of themselves, advanced by right of her completed "training," and, possibly, by some special aptitude for ruling and teaching nurses, to her present post.

### Everybody's Opinion.

*[Correspondence on all subjects is invited, but we cannot in any way be responsible for the opinions expressed by our correspondents. No communications can be entertained if the name and address of the correspondent is not given, or unless one side of the paper only be written on.]*

### THE MATRONSHIP OF LONGTON COTTAGE HOSPITAL.

A "Medical Correspondent" writes: Answering an advertisement in The Hospital of June 25th, a friend of mine applied for the post of Matron at the Cottage Hospital, Longton, Staffs. Applications, with testimonials and photograph, had to be sent in by July 2nd. The applicants heard nothing until they received a copy of the enclosed circular, which you will notice is dated July 5th, containing a curt statement that the former Matron has withdrawn her resignation and been re-appointed. No apology, no explanation; the only recompense that the disappointed ones get is, firstly, the valuable information that there were fifty-four candidates; and, secondly, the thanks of Thomas Blair, who, I suppose, is the Secretary. Do you think it honourable or right that the Managers of this hospital, after throwing the appointment open, should re-elect the former holder of the post? Is it not most unfair to put candidates to the trouble and expense of applying for a position which is afterwards declared not to be vacant? I think an authoritative opinion from you on this point would be of great service in the future to us.

[This incident is, we believe, unique of its kind. We should be glad to have an explanation of the causes which led up to it. As it stands, it has proved nothing better than a hoax to fifty-four ladies, who, at least, deserved reasonable consideration, which they certainly have not received.—Ed. *T. H.*]

### Presentations.

DR. J. H. BRIDGES.—A very interesting presentation took place on Monday, July 4th, in the library of the British Medical Association, when a massive silver bowl, subscribed for by the Medical Officers of the metropolitan fever hospitals, district asylums, poor-law schools, and workhouses, was given to Dr. J. H. Bridges (Local Government Board Inspector), who is on the point of relinquishing his office. The Chairman—Dr. Adams Clarke, Medical Officer of Leavesden Schools—in making the presentation, paid a graceful tribute to Dr. Bridges' high qualifications, uniform courtesy, and ability, and expressed regret at his impending resignation. Dr. Clarke dwelt upon the material improvements which had resulted in poor-law schools and infirmaries as the direct outcome of the suggestions made by Dr. Bridges from time to time, instancing especially the action taken in regard to ophthalmia, and the establishment of the metropolitan infirmaries on their present administrative basis. Dr. MacCombie (of the South-Eastern Hospital), Dr. J. H. Robinson (of Mile End Infirmary), Dr. Elliot (of Caterham District Asylum), and Dr. J. F. Williams (of Newington Workhouse) bore testimony to Dr. Bridges' courtesy, kindness, and help. Dr. Bridges, after expressing his gratification for the handsome present he had just received, spoke of the numerous difficulties encountered when he assumed the duties of an Inspector of the Local Government Board twenty-three years ago. He traced step by step the history of those important advances in general medicine and hygiene which had resulted from the work done in schools and workhouses; and he foreshadowed the Poor-Law infirmaries of the future, expressing the confident opinion that those institutions would be largely utilised for the purposes of medical instruction. A step in this direction had already been taken in opening the fever hospitals to students. In taking leave of those present, Dr. Bridges expressed the hope that he should not lose touch of those medical officers with whom he had been in contact for so many years. The proceedings were brought to a conclusion with formal votes of thanks to the Chairman, and to the Secretaries (Drs. Litteljohn and Sydney Stephenson) of the movement.

VENTNOR CONSUMPTION HOSPITAL.—On leaving the General Hospital to take up the post of Matron of the Ventnor Consumption Hospital, Miss L. J. Busby (who had been Matron for seven years) was presented with a handsome fumigated-oak writing table, bearing a brass plate: "Presented to Miss Louisa J. Busby by the nursing staff of the General Hospital, Birmingham, 24 June, 1892," and an album containing a list of the nursing staff, which included several former nurses.

### Appointment.

[It is requested that successful candidates will send a copy of their applications and testimonials, with date of election, to THE EDITOR, The Lodge, Porchester Square, W.]

BERKELEY HOSPITAL.—Miss [Caroline Fishwick has been appointed Matron of this hospital, out of forty applicants to the post. Miss Fishwick trained at the "London," and was afterwards appointed staff nurse in one of the men's surgical wards there; she afterwards went as accident nurse to the West Norfolk and Lynn Hospital, and subsequently took charge of the men's medical and surgical wards, and did a great part of the dispensing. For the last sixteen months Miss Fishwick has been Night Superintendent at the Bristol General Hospital.

### Wants and Workers.

HELP WANTED FOR VANCOUVER.—A lady is sending a parcel to Sister Frances, of St. Luke's Home, Vancouver, on August 1st, and will be glad if any readers of THE HOSPITAL will send her clothing to be included. Any sort of pretty or useful garments, new or old, will be welcome. Will some of those who have met Sister Frances or read of her work kindly answer this appeal?—Address *Mrs. Wm. Goslett*, 5, *Clifton Terrace, Brighton.*

### "LIGHT 'MID THE ENCIRCLING GLOOM."

IN our darkest hours, when amid doubts and perplexities we are feeling "As a child new born, it's mother dead, it's father far away beyond the seas," we blindly stretch out our arms and seek for Him who is surely hard by, though we see Him not. And though we grope like blind men and stumble at the obstacles in our path, yet our strong crying, our intense longing for light, which is in itself a sort of prayer, will in the end solve our difficulties, and bring their reward by showing the way plain before our face. At eventide there shall be light. Unfortunately, instead of waiting in faith and hope for the first streak of dawn we light our own farthing rushlights, and, the pity of it, even follow the false lights, the will-o'-the-wisps, which only lure us on to destruction. There is but one true Light, and that always shineth in darkness, though the darkness comprehendeth it not.

Work, however, is a lantern we may light with safety, and if the taper we burn in it be good, pure wax, it will give a steady flame, and cheer us very much in the dimness. It would be a poor substitute for the True Light if we depended only on it, yet it will help us to feel our way out of the gloom towards it. Christian and heathen philosophers alike agree in considering work the key-stone of existence.

There is no occasion to go out of the way to seek work: the daily round, the common task will be sufficient occupation for each person. Some have to work with their hands, others with their heads, but whether we write the grandest poem or only sweep a room, we must put our hearts into it and do it conscientiously, as that alone will make our actions fine. "Whatsoever thy hand findeth to do, do it with thy might" is an excellent motto for us all.

Alas, for those to whom doubt comes with suffering, sore indeed is the struggle. But they have work which they may do, patient waiting. It is as much work to bear pain with patience, to curb the sharp speech, to check the pettish action, when we are tied down to a sick bed as to make a steam-engine or sew a frock. And to such persons numerous promises are scattered up and down the Sacred Book. They are to have their heart's desire, to mount up with wings as eagles, to run and not be weary, to walk and not be faint.

Like the eagle, we must look up. The light does not come out of the ground but from heaven. If we look down we only see our own sores and weakness and misery; but if we look up we forget our troubles, at least for a time, and by watching earnestly for the day star to arise we shall be prepared to hail with joy the Sun of Righteousness which shall arise with healing in His wings. God led His people through the wilderness as safely by the cloud in the daytime as by the light of fire at night. Let us then take the comfort which the patience of hope gives, and be sure that He will lead us on to "Light's abode, celestial Salem."

### Off Duty.

### The Medicine Boy.

He was, there is no denying, a man with a very hasty temper, but none of his patients knew it; the ladies one and all declared that he was the very nicest doctor, and the men called him an uncommonly good fellow. It was universally conceded in the neighbourhood that illness lost half its terror if one were fortunate enough to secure the services of Dr. Bark. Nobody, indeed, ever saw anything of his tempestuous nature, except John, his coachman, and Sam, the medicine boy. The coachman was tall, lean, and bony; the medicine boy was short and stout, and to all appearances, boneless, and the buttons on his coat far exceeded those of the coachman in number and brilliancy.

The coachman was thirty years of age, the medicine boy only thirteen; but sharpness does not always depend upon age, and in point of sharpness the medicine boy could give the coachman points. Both had much time for reflection, but the coachman read penny novelettes, while the medicine boy hatched plots. You did not always suspect him of doing so, because, as a rule, great depth of expression lies in the human eye, and you can detect signs by carefully watching the same. With the medicine boy this was not, however, the case; he squinted, and you cannot ever expect to fathom the moral obliquity of a person whose vision is also oblique. Besides, the medicine boy was uncommonly deep and kept his counsel; to use his own metaphor, "You only know that the stuff inside a bottle is safe when you've rammed the cork in yourself." The coachman, on the contrary, did not bottle up his sentiments; he was tired, he said, of being bullied and sworn at, he was tired of being told that he was of no more use than a post, he was above all tired of being ordered to drive to a place where he prayed and hoped that he might never have occasion to go. The medicine boy looked furtively round; the two were alone in the stable, there was no chance of being overheard. He took heart of grace and responded to this outburst by another equally unpremeditated and quite as eloquent. He acknowledged, albeit with bated breath, that there were things of which he also had wearied. Like his friend, he was tired of being bullied and sworn at; like his friend, he was tired of being worked hard, and told that he was of no use; furthermore, having no opportunity of driving to the obnoxious destination already referred to, he was tired of being told to repair thither upon his two stumpy, wearied legs. He announced his intention of revolting. In this particular, and in the dogged courage of his demeanour, he differed from his friend.

"What are you going to do?" asked the coachman.

"I dunno," replied the medicine boy; "I'm a going to think it over. Just you hold on a bit. I'll let you know before you are many days older."

The coachman held on for a good many days, during which period he cheered his soul with the lightest of fiction, while the medicine boy went about his duties in a sullen way, and the doctor conducted himself as he had conducted himself for many years past—winning the love, admiration, and respect of all but his two hirelings.

Late one night, when the coachman was in a mood of the deepest despondency, the medicine boy surprised him in their old rendezvous, the stable. He was rubbing down his horse, to the usual hissing accompaniment set to a minor and melancholy key.

"He's been a going on awful, ain't he?" asked the medicine boy in a dark and mysterious whisper.

"Yes," replied the coachman, "I'm pretty nigh sick of it, I am."

The medicine boy established himself upon an elevated seat, and sat there, swinging his legs rythmically to and fro. Now and then he indulged in a low and portentous whistle.

"I've got something to say," he remarked, "and I'll say it as soon as you are ready, not before."

This was a gentle reminder that the coachman was slow in his movements, and served to accelerate them slightly. When Peggy, the mare, was duly disposed of, he condescended to reveal his plan of revenge; it was neat, deliberate, and so bold that it fired the coachman with enthusiasm. He became at once conspirator number two, and they put their heads together. Two heads might have been seen in deep deliberation, the coachman sleek and neatly trimmed, the youthful villain's shaggy red and abundant in supply. Whether or no the plot would be carried out next day depended on a certain contingency.

"If he tells me to drive to the devil, then I'm game," said the coachman.

"If he cuts up rough, and says I may go to the devil with the physic bottles, you shall see what you shall see," affirmed the medicine boy.

Next day the doctor kept his temper, but in the evening it escaped him. So did the objectionable phrase. The coachman heard it first, and winked at the medicine boy from the box; the medicine boy heard it second, and winked at the coachman from the pavement.

The doctor had just dined, and was vexed to be called out again. He was invariably sleepy after dinner, and a dense fog added to the discomfort of the situation. But his coachman was reliable, he could therefore close his eyes and indulge in a comfortable snooze.

He slept long and peacefully—he woke to find himself in the centre of a large and lonely common; one man held his horse's head, and a second held a lantern to the doctor's face, but John, the coachman, was nowhere to be seen. A paper was stuck in the carriage window, and the legend ran thus:—

"You said as how I was to drive you to the devil, and I've done the best I could.—Yours truly, John."

The doctor pondered for a moment, then got up on the box and drove home through the foul-smelling fog. When he entered his surgery what a sight met his eye. Neatly ranged upon a table stood the day's orders for medicine, lotions, tonics, many urgent prescriptions—none delivered to their destination. Beside them, written in a round, boyish hand, a large placard lay. It was as follows:—

"I couldn't take these bottles to the devil, 'cos I didn't rightly know where he lived.—Yours respectfully,
"Sam."

### Notes and Queries.

### Answers.

*Ima.*—Three hundred pounds will found a cot during the lifetime of a donor, and a thousand pounds will found one in perpetuity. The donor can always have one child in the hospital and a good supply of out-patients' letters.

*Patience.*—We can hear of no institution which would send you out as you suggest. How would it be to advertise?

*Francesca.*—Unless you have any influence in the countries you name you would have but little chance of an appointment. America trains its own nurses now, and the Colonies do too. Do you know anybody in Cape Colony that would be the most likely, and you mention that as a place you had thought of?

*A. B. C.*—The ladies who desire to become Her Majesty's nursing sisters must first be trained three years in a good general hospital. For full particulars apply to the Director-General, Army Medical Department, Victoria Street, Westminster.

*Vincent.*—The main building of the Ramsay Hospital, Naini Tal, is devoted to Europeans and Eurasians, containing 12 wards. The wards for native male patients, containing eight separate rooms and two general wards, with six beds each, or a total of 20 beds, are in a separate building. The native female wards are again in a separate building, with a total of 16 beds. It will have been formerly opened a year in October. Miss Georgina Macvitee is the Lady Superintendent.

## Figures and Tables

**Figure f1:**
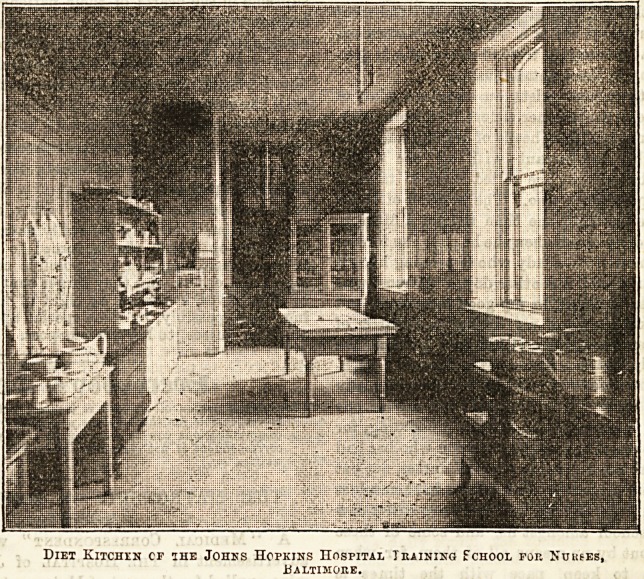


**Figure f2:**